# Frozen Microemulsions for MAPLE Immobilization of Lipase

**DOI:** 10.3390/molecules22122153

**Published:** 2017-12-05

**Authors:** Valeria Califano, Francesco Bloisi, Giuseppe Perretta, Antonio Aronne, Giovanni Ausanio, Aniello Costantini, Luciano Vicari

**Affiliations:** 1 Istituto Motori-CNR, via G. Marconi 8, 80125 Napoli, Italy; v.califano@im.cnr.it (V.C.); g.perretta@im.cnr.it (G.P.); 2 CNR-SPIN and Department of Physics “Ettore Pancini”, University of Naples Federico II, Piazzale V. Tecchio 80, 80125 Napoli, Italy; ausanio@unina.it (G.A.); vicari@unina.it (L.V.); 3 Department of Chemical Engineering, Materials and Industrial Production, University of Naples Federico II, Piazzale V. Tecchio 80, 80125 Napoli, Italy; anaronne@unina.it (A.A.); anicosta@unina.it (A.C.)

**Keywords:** MAPLE, microemulsion, lipase, thin film, enzyme immobilization

## Abstract

*Candida rugosa* lipase (CRL) was deposited by matrix assisted pulsed laser evaporation (MAPLE) in order to immobilize the enzyme with a preserved native conformation, which ensures its catalytic functionality. For this purpose, the composition of the MAPLE target was optimized by adding the oil phase pentane to a water solution of the amino acid 3-(3,4-dihydroxyphenyl)-2-methyl-l-alanine (m-DOPA), giving a target formed by a frozen water-lipase-pentane microemulsion. Fourier transform infrared (FTIR) spectroscopy and atomic force microscopy (AFM) were used to investigate the structure of MAPLE deposited lipase films. FTIR deconvolution of amide I band indicated a reduction of unfolding and aggregation, i.e., a better preserved lipase secondary structure in the sample deposited from the frozen microemulsion target. AFM images highlighted the absence of big aggregates on the surface of the sample. The functionality of the immobilized enzyme to promote transesterification was determined by thin layer chromatography, resulting in a modified specificity.

## 1. Introduction

Lipases (E.C.3.1.1.3) are ubiquitous enzymes able to catalyze reactions of large industrial interest, such as hydrolysis, esterification and transesterification of long-chain acylglycerides. In particular, the enzymatic transesterification of vegetable oil into biodiesel allows treating raw materials with high content of free fatty acids (i.e., waste fried oil), enabling the use of low quality, reused, and inedible oils without a negative impact on the environment [1]. The widespread application of lipase in industry, however, is inhibited because milder, eco-friendly, highly active, and selective enzymatic catalysis is not competitive with chemical catalysis. This is due, on the one hand, to the lack of operational stability (low stability to temperature and pH, loss of catalytic activity after one cycle of reaction), and on the other to difficult recovery and recycle of the enzyme [2,3,4]. These aspects are fundamental for industrial applications as they reduce process costs, offsetting the high production costs of enzymatic catalysts. Immobilizing enzymes on insoluble supports can address this issues [5]. Enzymatic immobilization allows combining the advantages of heterogeneous catalysis to an increased stability and sometimes increased activity [6] of the enzyme. It is therefore possible to facilitate the recovery and reuse of the catalyst and its separation from the reaction medium, lowering protein contamination of the reaction products. Immobilization often results in increased stability toward denaturation induced by heat, organic solvents, or inhibitors. This enhancement of the enzyme stability depends on the type of support and the technique used for the immobilization and can be due to a different microenvironment to which the immobilized enzyme is exposed and/or an enhanced rigidity of the immobilized enzyme [7,8]. Another important aspect, relevant for enzymes such as lipases (to which is often required to work in organic solvents), is that their immobilization may allow the enzyme to exert its catalytic activity in environments fundamentally different from the physiological one, i.e., in a non-aqueous solvents [3].

Matrix assisted pulsed laser evaporation (MAPLE) is a pulsed laser technique for thin film deposition, in which the target is composed of a frozen solvent (the matrix) containing a low volume fraction of the material to be deposited (the guest material). The matrix is a volatile, light absorbing solvent. When a laser beam impacts the target, droplets of both the volatile solvent and the solute are ejected away from the target. A vacuum system pumps away the matrix molecules, while the guest material deposits onto the substrate. The matrix absorbs most of the laser radiation giving a softer ejection mechanism with respect to pulsed laser deposition (PLD), hence transferring delicate molecules from the target to the substrate in undamaged form [9,10,11]. Due to such attributes, the MAPLE technique has been successfully used to deposit several proteins in an undamaged and functional form [12,13,14,15]. However, the MAPLE deposition of lipase is a sensitive issue, since lipases are unique enzymes in that most of them, including *Candida rugosa* lipase, require interfacial activation for full catalytic performance [16]. Actually, since lipids are water insoluble, lipases act on emulsified systems. Upon adsorption at a hydrophobic/hydrophilic interface, lipase undergoes a conformational change from the inactive to the active conformation. This change is promoted by the movement of a helical loop from the “closed” form in which the catalytic site is inaccessible to the “open” active one. During the immobilization procedure the mechanism of interfacial activation must be preserved and lipase is preferably to be obtained in the “open-lid” conformation, known to be more active [17,18,19,20].

Solvent assisted techniques for lipase immobilization affect its conformation by both the nature of the substrate and the adsorption conditions (i.e., pH of the solution). Benefits of using MAPLE for lipase immobilization include independence of lipase conformation on enzyme/support interaction, dry deposition allowing the use water-incompatible supports, and the possibility of tailoring lipase conformation in solution. *Candida rugosa* Lipase (CRL) films were already obtained by MAPLE using a water matrix [21], but conformational analysis of the obtained films pointed out an important protein unfolding and aggregation/self-association.

In this work, CRL was deposited by MAPLE improving the matrix composition by adding m-DOPA and pentane to the water matrix to obtain lipase films in which the secondary structure of the enzyme was better preserved. We show that the addition of an oil phase to the MAPLE target led to a better preservation of the protein secondary structure. Pentane was intended to induce lid opening, stabilizing the active conformation of lipase. Immobilization of lipase in the active form was immobilized by adsorption on a hydrophobic support favoring the open form of lipase in solution by varying the medium conditions [22].

m-DOPA (3-(3,4-dihydroxyphenyl)-2-methyl-l-alanine) is an analogous of L-DOPA (3,4-dihydroxy-phenyl-l-alanine) with a methyl group added. L-DOPA is an unusual amino-acid residue found in the adhesive protein secreted by mussels and responsible for mussel fouling on a variety of surfaces [23]. The catechol side chain of L-DOPA is thought to be responsible for the adhesive role of these proteins. The use of MAPLE for the deposition of m-DOPA was already discussed for improving polyethylene glycol (PEG) adhesion in anti-biofouling applications [24]. m-DOPA was added to accomplish three roles: (1) to absorb the laser radiation, reducing the risk of photochemical damage to the protein; (2) to protect lipase against unfolding/aggregation that can occur during drying in plume expansion [25]; and (3) to improve adhesion of the protein to the substrate. Actually, this aminoacid adheres to various surfaces via different types of interactions, including hydrogen bonds, metal coordination, electrostatic interaction, and so on [26]. It can bind proteins with either weak interaction such as hydrogen bonding or with covalent bonding [27], thus creating a bridge between the bound polypeptide and the grafted substrate.

## 2. Results and Discussion

The MAPLE deposition parameters are reported in Table 1.

The choice of the laser wavelength was set by the need of inducing as little damage as possible to the protein, since lipase has a large absorption band, centered at 280 nm [28], in the UV region normally used for MAPLE deposition and a N-H stretching vibration absorption in the IR region used for resonant infrared MAPLE (RIR-MAPLE, 2900 nm) [29]. Our focus was the deposition of lipase with preserved tertiary structure, since in catalytic applications obtaining a smooth deposition is not essential. This suggested us to select a small substrate to target distance in order to maximize the deposition yield.

Samples were obtained by varying the composition of the target solution, adding the excipient m-DOPA and the apolar solvent pentane to the lipase/water matrix system. The target composition and laser pulse energy used for each deposition are reported in Table 2. The pulse energy used corresponded to the minimum value allowing target evaporation. One of these samples, M-CRL1, was already characterized in a previous work [21].

m-DOPA was added because it absorbs the IR laser radiation, since the third overtone of C-H stretching of aromatic groups is located around 1064 nm [30], while water does not have absorption bands near that wavelength. Actually, m-DOPA lowered the ablation threshold of the target thus allowing using lower laser pulse energy, as indicated in Table 2. Furthermore, m-DOPA acts as an excipient to protect lipase from denaturation induced during plume expansion by drying [28] and improves adhesion. Pentane was added to induce lid opening and was responsible for the microemulsion formation. The addition of pentane, 0.5% in volume respect to the water, did not cause any phase separation or opalescence. Since pentane is poorly soluble in water (solubility at 25 °C in molar fraction *x* = 1.2 × 10^−5^ [31] vs. *x* = 7.8 × 10^−4^ used in this work), we assume that a microemulsion was formed due to the amphiphilic nature of lipase [32]. Proteins can be said to be more interfacial active than low molecular weight surfactant [33]. The tendency of lipases (and many other proteins) to interact with hydrophobic surfaces is a consequence of the amphiphilic character of the macromolecules. The mechanism of the pentane/water/CRL microemulsion formation of this study is not known. However, a hypothesis is that in the water solution of lipase couples of open lid molecules may interact with each other via the large hydrophobic surface surrounding the active center, creating a hydrophobic pocket. Palomo et al. [34] in fact proposed that some lipases, including CRL, may have a specific trend to self-assemble even in diluted solutions giving bimolecular structures formed by two open lipase molecules interfacially activating each other. MAPLE deposition from a frozen microemulsion target was feasible since protein stabilized emulsions, unlike other emulsified systems, have reduced tendency to phase separation during freezing [35].

**Figure 1:**
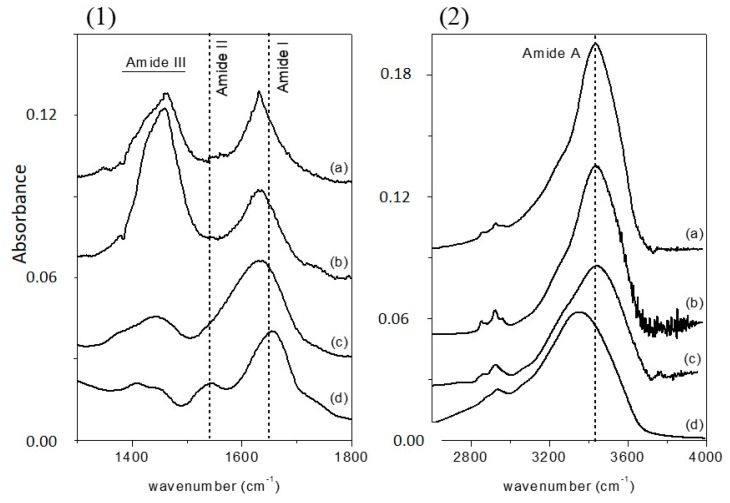
Fourier transform infrared (FTIR) spectra of (a) M-CRL1; (b) M-CRL2; (c) M-CRL3; and (d) free-CRL in the wavenumber range 1300–1800 cm^−1^ (**1**) and 2600–4000 cm^−1^ (**2**). The spectra were normalized to the height of amide I absorption band.

Infrared spectroscopy is a well-established technique for the conformational analysis of proteins [36] and has been used to study the secondary structure of proteins both in water solution [37,38] and in the solid state [39,40]. Fourier transform infrared (FTIR) spectra of the three MAPLE-deposited samples, together with those of free-CRL, are reported in Figure 1a (1300–1800 cm^−1^ range) and 1b (2600–4000 cm^−1^ range), since the absorption of the various secondary structural elements of the protein backbone can be studied in these spectral regions. The spectra were normalized for the height of the band at 1655 cm^−1^. In Figure 1, the FTIR spectrum of free-CRL (trace d) shows four main absorption bands ascribed to the vibration modes of the protein backbone. The band in the 1600–1700 cm^−1^ region, the amide I absorption band, centered at 1655 cm^−1^, originates mainly from the C=O stretching vibration of the peptide group, whose frequency depends on the strength of hydrogen-bonds C=O---H-N and on the dipole-dipole interactions between carboxyl groups along the peptide chain. In turn, this is determined by the secondary structure adopted by the polypeptide chain and this band is therefore sensitive to the protein conformation [37]. This band consists of overlapping components representing secondary structure elements such as α-helices, β-sheets, β-turns, and disordered structures. Several methods have been developed to estimate quantitatively the relative contributions of different types of secondary structures. Among these, second derivative spectra allow the identification of various secondary structures present in the protein. Curve fitting procedure can then be applied to calculate quantitatively the area of each component representing a type of secondary structure. The band centered at 1540 cm^−1^, amide II, is mainly related to the out-of-phase combination of the N-H in-plane bending and the C-N stretching vibration. This band also strongly depends on the secondary structure elements from which it originates, but the correlation structure/frequency is less straightforward. The amide III (1370–1480 cm^−1^) derives mainly from the in phase combination of the N-H bending and the C-N stretching vibration; due to the complexity of these contributes there is not a direct correlation between the position of this band and the protein structure. The band in the range 3420–3450 cm^−1^, the amide A absorption band, is due to the stretching of the N-H bond. The frequency of the amide A vibration depends on the strength of the hydrogen bond. In particular, N-H bonds in α-helices and β-sheets absorb around 3300 cm^−1^, while in random-coil structure the absorption band shifts to 3400 cm^−1^ [41].

By comparing the spectra of free-CRL and MAPLE deposited lipases (Figure 1), three main differences were seen: in the spectra of MAPLE deposited lipases (i) the amide I band shifted towards lower wave-numbers; on the contrary; (ii) the amide A band shifted to higher wave-numbers. Moreover, and (iii) the relative intensity of the amide II band was strongly reduced resulting partially overlapped with the amide I band for the M-CRL2 and M-CRL3. Further differences are seen in the 1300–1500 cm^−1^ region: in the spectra of M-CRL1 and M-CRL2, a strong band centered at 1460 cm^−1^ occurred, whereas in the spectrum of M-CRL3, a weaker and smoother band was seen that resembles the envelope of the two bands occurring for the free-CRL.

**Figure 2:**
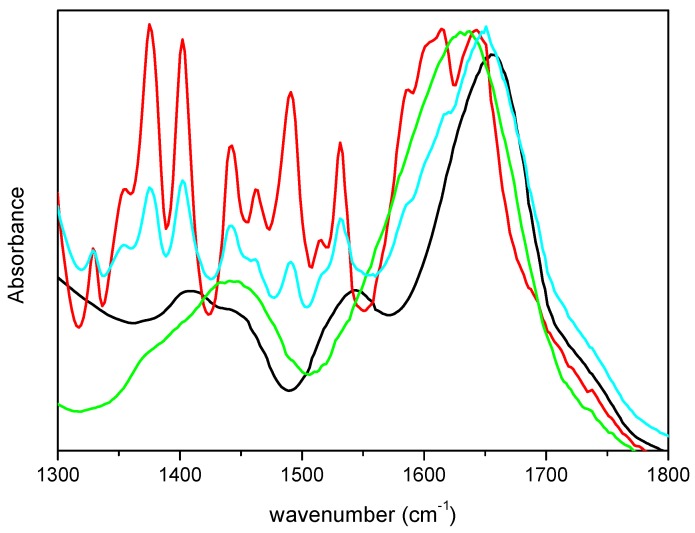
FTIR spectra of m-DOPA (red), free-CRL (black), M-CRL3 (green), and CRL/m-DOPA 1.8 mg/0.6 mg diluted in KBr (cyan).

To ascertain that the differences observed in the spectra were not due to vibrational modes of m-DOPA, its FTIR spectrum was acquired and is showed in Figure 2, together with the spectra of lyophilized CRL and M-CRL3 for comparison. Furthermore, a mix of 1.8 mg of CRL and 0.6 mg of m-DOPA (three times the amount used in the MAPLE target) diluted in KBr (total weight = 200 mg) was also examined, to see how the presence of DOPA would have influenced the spectrum of CRL, and is also reported in Figure 2. It is evident that the bands of m-DOPA were not visible in the spectra of MAPLE deposited samples, since they would have appeared as quite narrow peaks in the 1300–1550 cm^−1^ range, as illustrated in the spectrum of the CRL/m-DOPA mix. Furthermore, despite the fact that the amount of m-DOPA in the mix was thrice with respect to the MAPLE target, the displacement of its amide I band was much smaller respect to that of M-CRL3 (6 cm^−1^ vs. 23 cm^−1^), confirming that the differences observed in the spectra of the samples were due to the CRL conformational changes and not to the presence of m-DOPA. Finally, the spectrum that presented the major differences was the one of the sample M-CRL1, were m-DOPA was absent.

**Figure 3:**
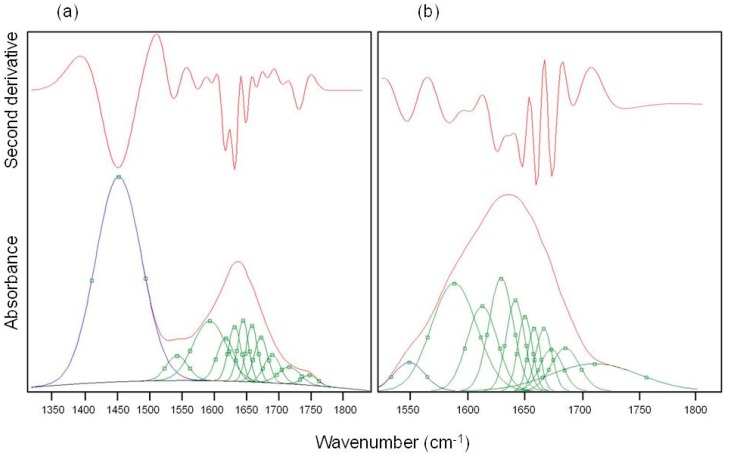
Best fit Gaussian components (green) and second derivative spectra (red) of amide I of (**a**) M-CRL3 and (**b**) M-CRL2. The blue peaks belong to amide II.

The displacement of the amide I band towards lower wave-numbers was already attributed to an important phenomenon of protein unfolding/aggregation [21]. From the spectra of Figure 1 it can be seen that this shift was more pronounced in M-CRL1 and M-CRL2 (about 27 cm^−1^) than in M-CRL3 (about 20 cm^−1^). To determine the degree of unfolding, a curve fitting procedure was applied to the spectra of the three MAPLE deposited samples and compared with that of free lipase. The results are reported in Table 3 while in Figure 3 are illustrated the second derivative spectra of the amide I used as fit initial values and the best fit Gaussian components obtained for M-CRL2 (trace (b)) and M-CRL3 (trace (a)). Since in these spectra the amide I and amide II bands were partially overlapped, in the fitting procedure, the amide II was considered as a component of the amide I band. Moreover, in the spectrum of M-CRL2 the amide III band was partially overlapped with the amide II. Consequently, to avoid errors in the baseline correction, these two bands were included in the fitting procedure.

The Gaussian components at wavenumbers smaller than 1590 cm^−1^ were attributable to components of amide II band overlapping with amide I, while the component occurring in the 1580–1590 cm^−1^ range was due to the C=O stretching of the COO^−^ aspartic and glutamic acid side chain. As matter of the fact, based on the values of lipase pKa, Asp and Glu side chains were largely deprotonated at the matrix pH (=7). The components at wavenumbers higher than 1610 cm^−1^ were related to the C=O stretching in the various conformational environment of the protein (α-helices, β-sheets, β-turns and disordered structures, intermolecular β-strands). From the area of each of these components, it was possible to evaluate the percentage of the secondary structure elements for the four samples. The results are reported in Table 4.

CRL conformation was determined by X-ray diffraction (XRD) on lipase crystals [44,45]. XRD data revealed in the CRL open conformation 30% for α-helices and 12% for β-sheets. In this study, the content of α-helices of free lipase was lower and that of β-sheets higher with respect to X-ray diffraction data. This is ascribable to the process of lyophilisation [46]. Proteins composed of α-helices and mixtures of α-helices and β-sheets have generally shown reversible conformational changes upon freeze-drying. Most commonly, a significant decrease in α-helix content was detected, with a simultaneous increase in β-sheet formation (possibly intramolecular in nature), as occurred in the free lipase sample under study. Furthermore, the presence of intermolecular β-sheets due to aggregation was detected (14.8% aggregates in free CRL). MAPLE deposited samples showed a decrease in the β-sheet content which was dramatic in M-CRL1 sample and attenuated going toward the M-CLR3 sample. Contextually, the content of α-helices fell. In M-CRL1 sample the content of intramolecular aggregates was 27.8%, but it fell at 19.6% in M-CRL2 sample, indicating that the addition of m-DOPA was effective in reducing the phenomenon of lipase self-association, likely by providing hydrogen bonds during the freezing of the MAPLE target and drying in plume expansion. Nevertheless, there was still a certain degree of unfolding, as testified by the high percentage of disordered structures comparable with those of M-CRL1 sample. The degree of unfolding decreased in the M-CRL3 sample, confirming that the formation of a microemulsion target was efficient in better preserving the polypeptide conformation during the MAPLE process.

This interpretation can be substantiated by the analysis of amide A band in the 3000–3700 cm^−1^ region. Curve fitting of the amide A band for the three MAPLE-deposited lipases was performed giving two Gaussian components for all samples (Table 5). In this region the contribute related to the O-H stretching of water can be considered to have a little influence in the overall absorption, since it was demonstrated that the MAPLE process is effective in removing water from the deposited samples [47]. The amide A band can be considered as due to the overlapping of two components: one at about 3300 cm^−1^ can be related to hydrogen-bonded N-H stretching vibrations; the second at about 3420 cm^−1^ can be related to vibrations of free N-H groups of the unfolded peptide forms [41]. Hence, the displacement of amide A band toward higher wavenumber can be related with unfolding of the polypeptide backbone.

The position of the two peaks was not the same in the four samples, since N-H vibration frequency will depend on the environment of the N-H bond. For example, the position of the hydrogen-bonded N-H stretching vibrations depends on the strength of the hydrogen bond [48]. It is worth noting that the positions of M-CRL3 peaks were closer to free-CRL respect to the other two samples.

Since N-H and N-H---O bonds have different dipole moments, resulting in different intensities of the respective absorption bands, the area of the peak 2 was not directly correlated with the degree of unfolding of the protein, even if its value decreased going from M-CRL1 to M-CRL3.

To give a quantitative measure of the unfolding occurring in the samples, the relative unfolding was expressed as percentage from the area of peak 1 assuming as a reference the value of free-CRL (Table 5). The percentage of unfolding in M-CRL2 was even a little higher than M-CRL1, while it was reduced by 23 percentage points in M-CRL3.

Finally, the band centered at 1460 cm^−1^ was also strictly related to the unfolding of the protein. N-H in-plane bending mode shifted downward in frequency with H bond breaking [49], superimposing with the modes of amide III, giving rise to a stronger absorption band centered at 1460 cm^−1^ and lowering the intensity of the amide II band. The intensity of the band at 1460 cm^−1^ was greatly reduced in the spectrum of M-CRL3, confirming that in this sample the protein conformation was better preserved. This may be due to the combined effect of decreasing laser power and the greater conformational rigidity of lipase adsorbed on the water/oil interface. Preserving the native conformation of lipase is important for it to perform its catalytic activity.

**Figure 4:**
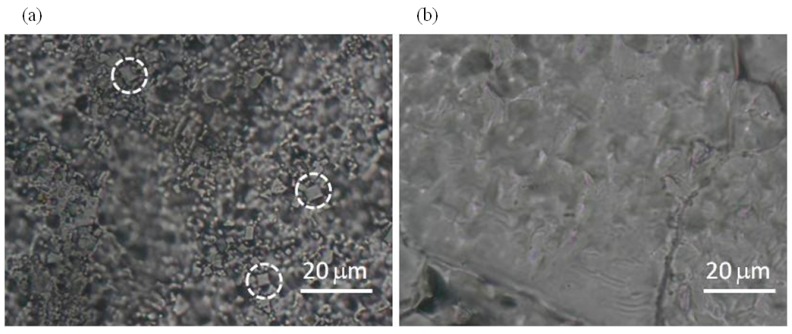
Micrographs of (**a**) M-CRL2 and (**b**) M-CRL3. White circles highlight crystalline inclusions.

In Figure 4, optical micrographs of M-CRL2 and M-CRL3 are shown. As displayed in Figure 4a, M-CRL2 film was formed by micrometric clusters and crystalline inclusions, some of which are evidenced by white circles. These features outline that m-DOPA was effective in absorbing the infrared laser radiation thanks to its catechol side chain, its addition to the matrix target causing the deposition of a larger mass of material at a lower laser pulse energy with respect to the sole lipase/ice target [21]. The formation of clusters is typical of the MAPLE process but does not implies that they are formed of aggregate molecules of enzyme. The formation of micrometric clusters evidenced in Figure 4 has often been observed in MAPLE deposition [24,50,51,52]. It depends on the explosive disintegration of the target hit by the laser that gives rise to the ejection of a mixture of vapor-phase molecules, small molecular clusters, and droplets [53]. The solvent begins to evaporate during the flight from the target, so that the solute concentration in droplets and clusters increases causing the deposition of micrometric clusters. The surface of M-CRL3 film, on the contrary, was much smoother with no visible clusters at the optical microscope, Figure 4b, indicating a better molecular dispersion of lipase, possibly due to its adsorption at the pentane nano-droplets of the microemulsion target.

**Figure 5:**
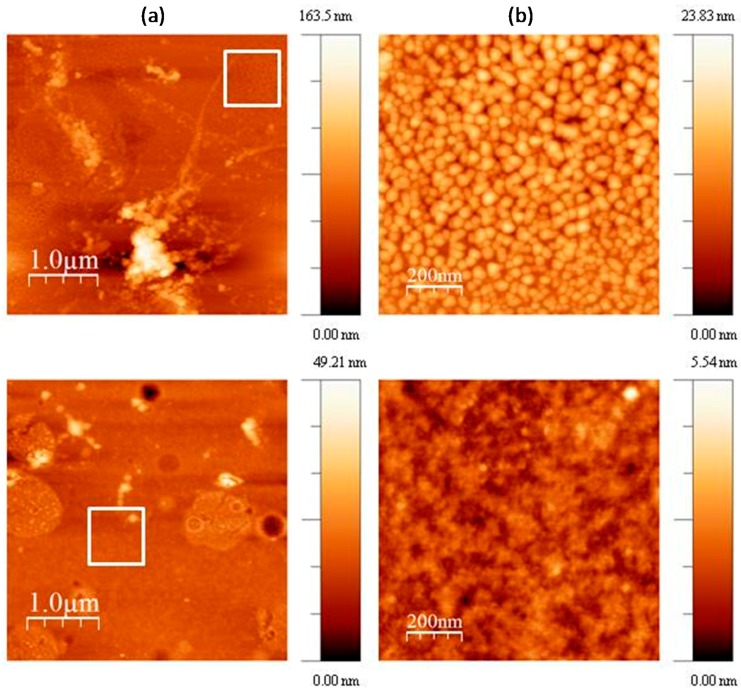
(**a**) 4 μm × 4 μm (left) and 1 μm × 1 μm (right) atomic force microscopy (AFM) images of M-CRL3 film; (**b**) 4 μm × 4 μm (left) and 1 μm × 1 μm (right) AFM image of M-CRL1 film. The white boxes indicate the regions where the magnification was performed.

To further investigate the morphology of M-CRL3, atomic force microscopy (AFM) images of the sample were acquired in different region of the samples. Some of such images are compared with the ones of M-CRL1 film [13] in Figure 5. Particularly, 4 μm × 4 μm (left) and 1 μm × 1 μm (right) AFM images of M-CRL1 film are displayed in Figure 5a, while 4 μm × 4 μm (left) and 1 μm × 1 μm (right) AFM image of M-CRL3 film are shown in Figure 5b. The high magnification images were taken in the region marked with a white box in the low magnification images. From AFM images of M-CRL1, it is evident that the substrate was uniformly covered by nanometric aggregates that were due to lipase self-association. These aggregates had mean planar dimension of 40 nm. Considering that lipase is a globular protein of approximately 5 nm in diameter, aggregates were composed of several lipase globules.

Figure 5b shows a different morphology for M-CRL3 film. As seen in the lower magnification picture, clusters were much smaller and concentrated in droplet-like regions of about ≤1 μm in size, while at the nanometric level the compact structure of aggregates of 40 nm was not present, instead much smaller features were detected from the higher magnification picture, confirming a reduction of self-association of proteins as already suggested by FTIR analysis.

**Figure 6:**
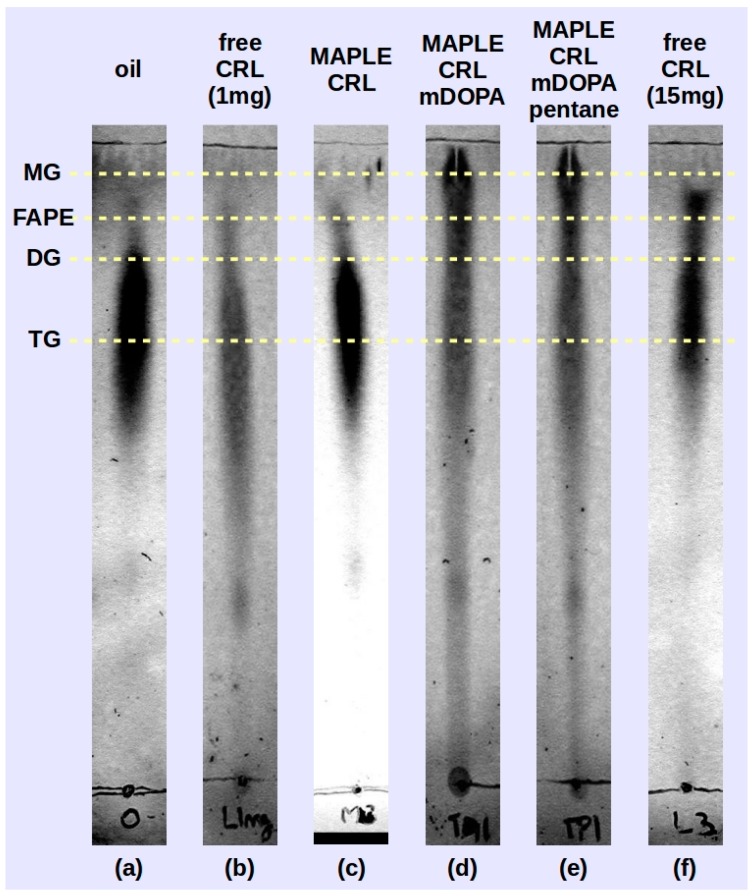
Thin layer chromatography (TLC) of transesterification products obtained the MAPLE deposited lipase M-CRL1 (**c**), M-CRL2 (**d**) and M-CRL3 (**e**) as biocatalysts are compared to the chromatogram of the unreacted oil (**a**) and that obtained using 1 mg (**b**) and 15 mg (**f**) of free-CRL as biocatalyst.

Preliminary tests of the deposited lipase samples were performed by transesterification reaction carried out between soybean oil and isopropyl alcohol using reverse phase thin layer chromatography (RP-TLC) to identify the reaction products. In Figure 6 the chromatograms of the three samples (M-CRL1 in Figure 6c, M-CRL2 in Figure 6d and M-CRL3 in Figure 6e) are compared with that obtained using 1 mg (Figure 6b) of free CRL as catalyst. The amount of 1 mg was chosen since this was the order of magnitude of the amount of lipase deposited by MAPLE. For the sake of clarity, the chromatogram of unreacted oil (Figure 6a) and that obtained using 15 mg of free CRL as catalyst (Figure 6f) are also reported. The amount of 15 mg was chosen to clearly show the action of lipase.

On the RP-TLC plate, the oil chromatogram shown in Figure 6a appeared elongated due to the variation in saturation and chain length of the component fatty acids [54]. For the reacted mixtures, the order of retention followed the polarity of the components: monoglycerides (MG) exhibited the least retention, followed by fatty acid propyl esters (FAPE), diglycerides (DG), and residual oil (TG, triglycerides) [53]. The RP-TLC results obtained using M-CRL1 as biocatalyst confirmed the data obtained with GC-MS analysis carried out in a previous study [21], allowing to identify peaks belonging to isopropyl esters of the most abundant fatty acid components of soybean oil, indicating that MAPLE deposited CRL preserved its functional role.

In the reacted mixture obtained using M-CRL2 (MAPLE deposited CRL with m-DOPA addition) and M-CRL3 (MAPLE deposited CRL with m-DOPA and pentane addition) lipase there was a higher quantity of MG and DG and a much lower quantity of FAPE with respect to the reaction mixture obtained with free-CRL. This occurrence appears unusual since CRL is a non-specific lipase, and in this kind of biocatalysts the reaction intermediates MG and DG do not accumulate since they react faster than the TG [55]. Actually, comparison of TLC analysis performed on the reaction mixture obtained using 1 mg (Figure 6b) and 15 mg (Figure 6b) of free-CRL shows that despite the smaller quantity of FAPE obtained, reaction intermediates DG and MG were not produced. We hypothesize that the higher production of MG and DG is due to the conformational changes induced in the lipase during the MAPLE process and evidenced by the FTIR analysis. Nevertheless, the presence of KBr as immobilization support is also important, since the presence of a salt can lead to the formation of fatty acid salts, which in turn may lead to enzyme inhibition through conformational changes induced by the electrostatic interaction between the anionic headgroup and sites of opposite charge on the protein [56].

## 3. Materials and Methods 

Lipase from *Candida rugosa* type VII (Sigma) with activity ≥700 U/mg solid (One unit U hydrolyzes 1.0 microequivalent of fatty acid from a triglyceride in 1 hr at pH 7.2 at 37 °C), and methyl-DOPA hemihydrate (Fluka) were acquired from Sigma-Aldrich (Milan, Italy). The composition of the lyophilizate is not specified, but it could contain surfactants, i.e., potassium sorbate [55], that can facilitate the emulsion formation. Deionized water was used to prepare MAPLE target solutions at pH = 7. All other solvents and compounds used were analytical grade from Sigma-Aldrich. Soybean oil was purchased in the local market. The MAPLE targets were prepared by dissolving the appropriate quantity of lipase in water (M-CRL1, 0.2 wt % of lipase), then adding the required amount of m-DOPA (M-CLR2, 0.18 wt % of lipase and 0.2 wt % of m-DOPA) and of pentane (M-CLR3 0.18 wt % of lipase and 0.2 wt % of m-DOPA and 0.5% *v*/*v* of pentane).

**Figure 7:**
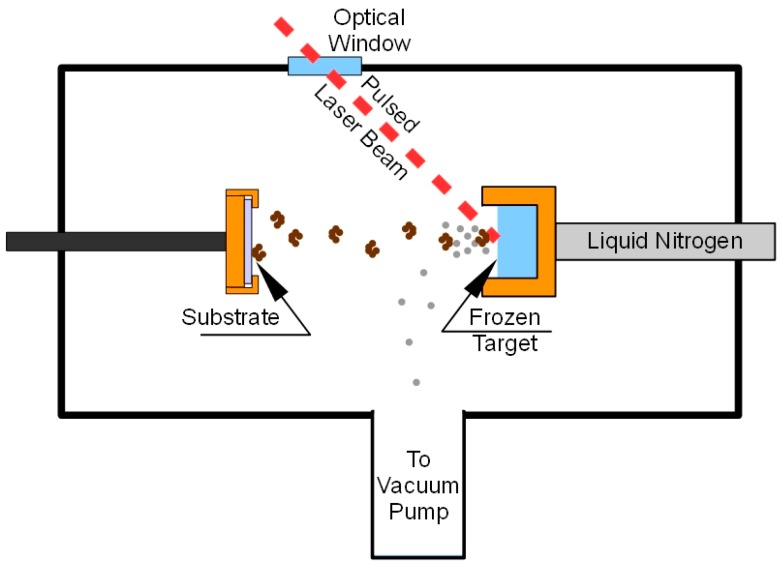
MAPLE deposition system.

A schematic of the MAPLE process is reported in Figure 7.

About 2 mL of the target solution were placed into the target holder and frozen by thermal contact with liquid nitrogen (−123 °C). The deposition chamber was evacuated (10^−4^ Pa), and afterward, the Nd:YAG pulsed laser was switched on to start the deposition. The target holder was moved by a computer controlled mechanical system so that the laser beam scanned an area of about 1.5 cm^2^ in order to prevent local overheating and drilling of the target. Lipase films were deposited on KBr pellets of 13 mm diameter in order to perform FTIR analysis. FTIR spectra were recorded, in the 4000–400 cm^−1^ range, using a spectrometer (Nicolet 5700, Thermo Fisher Scientific, Rodano, Italy) equipped with a DTGS KBr (deuterated triglycine sulphate with potassium bromide windows) detector. A spectral resolution of 2 cm^−1^ was chosen and each spectrum represents an average of 64 scans, corrected for the spectrum of the blank KBr pellet. For comparison, spectra were acquired also for lyophilized unprocessed lipase (free-CRL). Then, 4.0 mg of lyophilized lipase were mixed with 196 mg of KBr and pressed into pellets. The salt was previously dried at 100 °C for 24 h in order to eliminate the interference of water in the spectra.

The curve fitting of the amide I band was performed with curve-fit procedure of GRAMS/32, after smoothing and baseline subtraction. All spectra were analyzed by the second derivative method to determine the number of peaks and the peak positions to use as input parameters of the fitting. The second derivative spectra were obtained following the Savitsky–Golay method after a binomial 5 points smoothing of the spectrum followed by a baseline correction. The amide I band was analyzed in terms of a linear combination of spectral components identified in the second-derivative spectrum. Those components were approximated by Gaussian functions whose peak positions, widths and intensities were adjusted iteratively in the curve-fitting procedure. The initial full width at half height (FWHH) was 12 cm^−1^.

Images of the deposited CRL films on KBr substrate were obtained by means of an Olympus microscope with 100× magnification, using a micrometric slide to quantify the size of the aggregates formed in the process.

The typical sample morphology was determined by atomic force microscopy (AFM) analysis of deposits obtained onto KBr substrates. The analysis was performed by means of a Nanoscope IIIa AFM (Veeco Instruments Inc., Santa Barbara, CA, USA) operating in tapping mode (scan size and rate of 1 μm and 1 Hz, respectively) and equipped with a silicon tip having nominal curvature radius of about 5 nm.

In order to assess the ability of the deposited lipase to perform their catalytic role, transesterification reaction were carried out between soybean oil and isopropyl alcohol, using the surfactant span 80^®^ to emulsify the reactants. The reaction batches were prepared as follows: 0.043 g of Span 80^®^ were added to 1.7 g of soybean oil under stirring. Once the surfactant was dissolved, 0.7 mg of isopropyl alcohol were poured drop by drop in the solution, resulting in a slight opalescence (emulsion formation). To this reaction batches, CRL was added either in the free (1 mg and 15 mg) or in the immobilized form. The reaction mixtures were kept under stirring (200 rpm) for 24 h at 25 °C. The reaction products were qualitatively characterized by reverse phase thin layer chromatography (RP-TLC), using precoated TLC glass plates RP-18 (silica gel C18 layer of 0.25 mm, Macherey-Nagel (Düren, Germany) 5 cm × 20 cm in size as stationary phase and a solution of acetonitrile–ethylacetate 2.5:1 as mobile phase. The chromatograms were detected by spraying sulphuric acid 4 N on the chromatographic plates and heating them at 150 °C.

## 4. Conclusions

*Candida rugosa* Lipase (CRL) was deposited by MAPLE tailoring the target composition in order to minimize the conformational modification observed during the process. In particular, a phenomenon of unfolding/aggregation was evidenced. Experimental results evidenced that unfolding/aggregation induced by MAPLE process can be minimized by tailoring the target matrix.

FTIR analysis was performed to investigate the conformational changes that the enzyme underwent during the deposition process. From the deconvolution of amide I band of the samples, it resulted that in M-CRL1 sample (water matrix), the content of intramolecular aggregates was 27.8%, but it fell at 19.6% in M-CRL2 sample (water/m-DOPA matrix), indicating that the addition of the amino acid m-DOPA was able to prevent aggregation. From the analysis of amide A band, the percentage of unfolding in M-CRL2 is 65%, even higher than in M-CRL1, while it became 42% in M-CRL3 (water/m-DOPA/pentane matrix), indicating that the addition of pentane to form a microemulsion was effective for preventing unfolding. All MAPLE deposited samples showed catalytic ability, but reaction privileged MG and DG products differently with respect to free-CRL, which can be due to the MAPLE induced conformational modification of the deposited lipases together with the presence of inhibitors (fatty acid salts due to the presence of KBr as support material) or restraints to conformational changes in the enzyme due to immobilization. The addition of an apolar solvent to the MAPLE target attenuated the conformational alteration occurring during MAPLE process, which represents a feasible way to maintain unaltered the conformation of the enzyme during the MAPLE deposition.

## Figures and Tables

**Table 1:** Matrix assisted pulsed laser evaporation (MAPLE) deposition parameters.

Parameter	Value
Laser wavelength	1064 nm
Laser pulse energy	526–410 mJ/pulse
Pulse duration	7 ns
Pulse repetition rate	4 Hz
Number of pulses	23,000
Target-substrate distance	9 mm
Incidence angle	45°
Chamber pressure during deposition	10^−4^ Pa
Substrate	KBr
Target temperature	−123 °C
Substrate temperature	25 °C
Target matrix	distilled water

**Table 2:** Target composition of MAPLE deposited samples.

Sample	Target Composition			Pulse Energy (mJ/Pulse)
	Lipase	m-DOPA	Pentane	
M-CRL1	0.2 wt %	-	-	526
M-CRL2	0.18 wt %	0.02 wt %	-	410
M-CRL3	0.18 wt %	0.02 wt %	0.5% *v*/*v*	410

**Table 3:** Band position (cm^−1^) and assignment of the best-fit Gaussian components of the amide I band.

	Gaussian Component Position (cm^−1^)			Attribution	Literature Data
Free CRL	M-CRL1	M-CRL2	M-CRL3		
	1566	1539	1548	Amide II	1530–1550 [40]
	1585	1588	1587	C=O stretching of COO^−^	1565–1585 [40]
1609	1618	1613	1612	Intermolecular H-bonded C=O (aggregates)	1610–1620 [39]
1623	1631	1626	1629	β-sheets	1620–1650 [39]
1635					
1640	1642	1639	1641	Disordered structures	1640–1650 [42]
1650	1650	1652	1650	α-helices	1650–1660 [39]
1659	1657		1658		
1668	1667	1663	1666	β-turns	1660–1690 [39]
1676		1673	1673		
1685	1696	1687	1685	Β-turns/β-sheets	1680–1696 [42,43]
1711	1738	1710	1711	C=O stretching of -COOH	

**Table 4:** Percentage of the secondary structure elements.

Structural Element	Free-Lipase	M-CRL1	M-CRL2	M-CRL3
β-sheets	25.4	14.5	18.1	23.4
α-helices	27.2	21.3	15.6	15.4
β-turns	16.3	14.7	21.3	16.1
β-turns/β-sheets	13.7	4.3	8.1	10.1
Aggregates	14.8	27.8	19.6	21.1
Disordered	2.6	17.4	17.3	13.9

**Table 5:** Results of amide A Gaussian deconvolution: peak positions (cm^−1^), peak area, A, and relative unfolding evaluated as percentage from the area of peak 1 assuming as a reference the value of free-CRL.

Sample	Peak 1 Peak 2 Position (cm^−1^)	Peak Area A	Relative Unfolding %
M-CRL1	3222.5 ± 4.8	5.9 ± 0.29	65
	3451.5 ± 1.2	24.0 ± 0.29	
M-CRL2	3251.2 ± 6.8	4.9 ± 0.37	71
	3455.2 ± 1.8	19.8 ± 0.37	
M-CRL3	3322.7 ± 8.1	9.76 ± 0.68	42
	3494.8 ± 3.3	9.97 ± 0.67	
Free-CRL	3347 ± 4.8	16.7 ± 0.56	0
	3502 ± 3.6	2.1 ± 0.55	
